# DIDN’T SEE THIS COMING – DIAGNOSIS OF OCULAR SYPHILIS IN THE EMERGENCY DEPARTMENT

**DOI:** 10.51894/001c.122860

**Published:** 2024-08-30

**Authors:** Irene Young, Christopher Nedzlek

**Affiliations:** 1 Emergency Medicine Henry Ford Wyandotte

## 21

### INTRODUCTION

Ocular syphilis is an uncommon diagnosis, affecting approximately 0.15 per 100,000 populations and is not frequently identified in the emergency department (ED). It carries the potential for severe visual impairment if not promptly diagnosed and treated. This case details the presentation of ocular syphilis in an immunocompetent female diagnosed in the ED. The rate of primary and secondary (P&S) syphilis has steadily increased yearly since 2001, with a notable 28.6% rise from 2020 to 2021. Although syphilis rates are lower among women, they have surged significantly, increasing 55.3% from 2020 to 2021 and 217.4% from 2017 to 2021.

### CASE DESCRIPTION

36-year-old female presented to our community ED with a vague complaint of bilateral, painless, blurry vision. The patient’s history and physical exam revealed heterosexual activity with one male, tongue and genital ulcers, bilateral corneal abrasions, and a non-focal neurologic examination. Visual acuity was 20/400 bilaterally. The patient was tested and treated empirically for gonorrhea and chlamydia (G&C), had syphilis serology sent, and was discharged home with ophthalmology follow-up. The following day, the rapid plasma reagin (RPR) resulted positive, and the patient was called back to the ED for further evaluation and treatment. The patient underwent lumbar puncture (LP) to rule out neurosyphilis, which was negative and Venereal Disease Research Laboratory (VDRL) pending. G&C were negative. The patient received 4 million units of penicillin G and was transferred to a tertiary center for ophthalmology evaluation. After evaluation, they agreed findings were indicative of ocular syphilis, specifically uveitis. Per Centers for Disease Control and Prevention (CDC) guidelines, ocular syphilis is treated as neurosyphilis, with 14 days intravenous penicillin G, irrespective of LP results.

### DISCUSSION/CONCLUSIONS

Syphilitic uveitis is not frequently in the differential diagnosis of ED Physicians for patients presenting with blurry vision. With the substantial increase in the incidence of syphilis, ED Physicians should include iritis, uveitis, or chorioretinitis as ocular manifestations of neurosyphilis in their differential diagnosis for patients presenting with blurry vision. This case underscores the importance of a thorough history, review of systems, and maintaining a broad differential to facilitate early diagnosis and treatment, preventing irreversible vision loss.

